# Detection of the Asian II genotype of dengue virus serotype 2 in humans and mosquitoes in Brazil

**DOI:** 10.1590/0037-8682-0439-2019

**Published:** 2020-04-22

**Authors:** Janaina Rigotti Kubiszeski, Carla Julia da Silva Pessoa Vieira, Sirlei Franck Thies, David José Ferreira da Silva, Eriana Serpa Barreto, Adriano Mondini, Roberta Vieria de Morais Bronzoni

**Affiliations:** 1 Universidade Federal de Mato Grosso, Instituto de Ciências da Saúde, Sinop, MT, Brasil.; 2 Secretaria de Estado de Saúde, Sinop, MT, Brasil.; 3 Universidade de São Paulo, Departamento de Ciências Farmacêuticas, Araraquara, SP, Brasil.

**Keywords:** Molecular epidemiology, Arbovirus, Phylogeny

## Abstract

**INTRODUCTION:**

DENV-2 is the cause of most dengue epidemics worldwide and is associated with severe cases.

**METHODS:**

We investigated arboviruses in 164 serum samples collected from patients presenting with clinical symptoms of dengue fever and 152 mosquito pools.

**RESULTS:**

We detected the Asian II genotype of DENV-2 in humans and mosquitoes. Our results confirmed the circulation of the Asian II genotype in Brazil, in addition to the prevalent Asian/American genotype.

**CONCLUSIONS:**

The detection of Asian II genotype of DENV-2 in mosquito pools collected in a forest park may be related to a spillback event of human dengue virus.

Dengue fever is endemic in Brazil, and several epidemics have been reported over the years. Although the four serotypes of dengue virus (DENV-1 to DENV-4) are widespread throughout the country, a particular serotype is generally responsible for the majority of cases during an outbreak. DENV-2 is the most frequent cause of dengue fever epidemics worldwide and is usually associated with severe disease cases. DENV-2 is divided into six genotypes: (i) Asian I, comprising strains from Thailand; (ii) Asian II, consisting of strains from the Philippines; (iii) Cosmopolitan, comprising strains from South and Southeast Asia; (iv) American, comprising strains from Central America; (v) Southeast Asian/American, comprising strains from Southeast Asia and Central and South America; and (vi) Sylvatic, comprising strains from West Africa and Southeast Asia^1^.

The American genotype was the predominant genotype in Latin America until the early 1980s, when it was replaced by the Asian/American genotype, which has been associated with severe disease. The DENV-2 Asian/American genotype caused major epidemics in Brazil in 1990, 1998, and 2007/2008^2^. Herein, we report the circulation of the Asian II genotype of DENV-2 in human serum and mosquitoes collected in surveillance studies conducted in urban areas of Sinop (11º50’53” S and 55º38’57” W), a municipality in the northern state of Mato Grosso, Southern Amazon, Brazil (Figure 1A).

**FIGURE 1:**
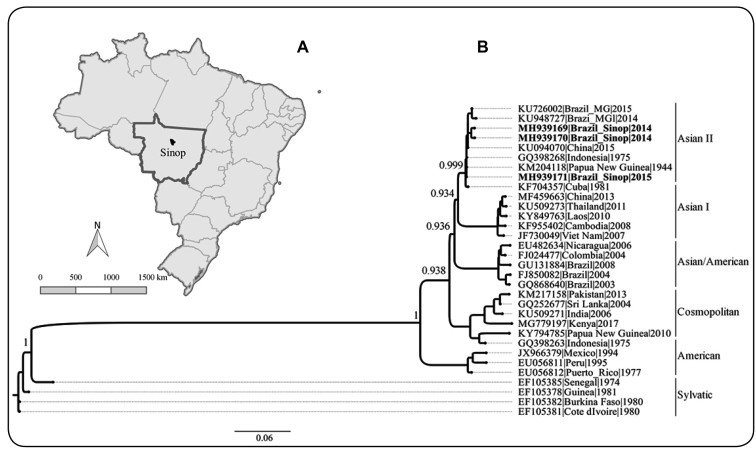
(A) Location of Sinop, in the state of Mato Grosso, Brazil. (B) The General Time Reversible (GTR) model with four gamma substitution rate categories (+ G, parameter = 0.203) (GTR + G) was used to reconstruct a Maximum Likelihood phylogenetic tree (PhyML) based on nonstructural protein 5 (NS5) partial gene sequences of the six DENV-2 genotypes. The sequences investigated in the present study are indicated in boldface. The SPR technique was used for the tree search operation, and the statistical support for the phylogenetic nodes was assessed using aLRT SH-like, which is indicated for each genotype. The final tree was edited in FigTree.

A total of 164 serum samples were collected from patients presenting with clinical symptoms of dengue fever between February 2014 and December 2015. Furthermore, 778 mosquitoes were collected using CDC light traps between May and October 2014^3^ (Table 1) and grouped into 152 pools. Collection sites were distributed throughout the city and included urbanized neighborhoods, neighborhoods near forest remnants and three areas of permanent preservation (Sinop Forest Park, Universidade Estadual de Mato Grosso - UNEMAT - Forest, and Horto Botanical Garden). Mosquitoes were grouped into pools of up to 20 specimens, according to species, collection site, and sex.

**TABLE 1:** Species of mosquitoes tested for arboviruses, collected between May and October 2014 in Sinop, Brazil.

Species	May	June	July	August	September	October	Total
*Aedeomyia (Ady.) squamipennis*	3 (F)	2 (F)	23 (F);	28 (F);			90
			14 (M)	20 (M)			
*Aedes (Stg.) aegypti*	1 (M)		2 (M)			2 (F);	7
						2 (M)	
*Coquillettidia (Rhy.) arribalzagae*	2 (F)						2
*Culex (Cux.) coronator*	14 (F);	34 (F);	3 (F)			3 (F);	66
	8 (M)	2 (M)				2 (M)	
*Cx. (Cux.) declarator*	4 (F);	13 (F)	5 (F)	11 (F);	64 (F);	20 (F)	124
	1 (M)			3 (M)	3 (M)		
*Cx. (Cux.) nigripalpus*	1 (F)	3 (F)	1 (M)	5 (F)	5 (F);		17
					2 (M)		
*Cx. (Cux.) quinquefasciatus*	66 (F);	6 (F);	15 (F);	66 (F);	13 (F);	8 (F)	261
	5 (M)	5 (M)	17 (M)	41 (M)	19 (M)		
*Cx. (Cux.) saltanenses*			13 (F)	8 (F);	8 (F)	11 (F)	41
				1 (M)			
*Cx. (Mel.) sp*		9 (F)	12 (F)	15 (F);	65 (F);	12 (F);	125
				1 (M)	5 (M)	6 (M)	
*Haemagogus (Hag.) tropicalis*						1 (F)	1
*Ochlerotatus (Och.) scapularis*		1 (F)	3 (F)	1 (F)	15 (F)	6 (F)	26
*Psorophora (Gra.) dimidiata*	1 (F)		1 (F)				2
*Uranotaenia (Ura.) ditaenionota*					1 (F)	4 (F);	6
						1 (M)	
*Ur. (Ura.) geometrica*		1 (F)	2 (F)	1 (F)	1 (F);	1 (F);	10
					3 (M)	1 (M)	
Total	106	76	111	201	204	80	778

(F) pools containing female specimens; (M) pools containing male specimens.

Viral RNA was extracted from serum samples and pools of homogenized mosquitoes using the QIAmp Viral RNA Mini kit (Qiagen GmbH, Hilden, Germany) and TRIzol reagent (Invitrogen; Thermo Fisher Scientific, Inc., Waltham, MA, USA), respectively. Samples were screened for arthropod-borne viruses of the genera *Flavivirus* and *Alphavirus* using Multiplex-Nested RT-PCR employing specific primers targeting regions of the nonstructural protein 5 (NS5) and nonstructural protein 1 (nsP1)^4^, respectively. We confirmed our PCR results by isolating the viruses in the Vero/E6 and *Aedes albopictus* C6/36 cell lines and subjecting them to multiplex-nested RT-PCR^5^. PCR products from serum samples, mosquitoes, or viral isolation were purified using the PCR Clean-Up System kit (Promega Corporation, Madison, WI, USA). Purified products were directly sequenced with species-specific primers, using the BigDye Terminator v3.1 Cycle Sequencing kit (Thermo Fisher Scientific, Inc.) and an ABI377 automatic sequencer (Applied Biosystems). Virus identity was determined using the Basic Local Alignment Search Tool (https://blast.ncbi.nlm.nih.gov/Blast.cgi).

We detected arthropod-borne viruses in 55 febrile patients. Two serum samples, collected in December 2015, tested positive for DENV-2 through multiplex-nested-PCR and viral isolation. One of the aforementioned samples (BR/Sinop/H361/2015) was isolated from a 13-year-old male patient presenting with headache and normal leukocyte (4,660/mm^3^) and platelet (157,000/mm^3^) counts. The other sample (BR/Sinop/H345/2015) was collected from a 47-year-old female patient presenting with headache, arthritis, itchiness, rash, and normal leukocyte (4,040/mm^3^) and platelet (332,000/mm^3^) counts. Interestingly, two pools of *Culex,* subgenus *Melanoconion,* collected in September 2014 tested positive for DENV-2 by multiple-nested-PCR, but not by cell culture. The mosquitoes were collected in a trap from a forest park area (11°50’0.0”S and 55°23’58, 3”W) in Sinop. The pools (BR/Sinop/Ar23/2014 and BR/Sinop/Ar6/2014) consisted of eight and five female specimens of *Culex sp*., respectively.

We obtained one NS5 partial gene sequence of DENV-2 from a serum sample (BR/Sinop/H345/2015; GenBank accession number MH939171), and two sequences from mosquitoes pools (GenBank accession numbers MH939169 and MH939170). Phylogenetic analysis based on the NS5 partial gene sequences of DENV-2 (316 base pairs) indicated that our sequences clustered within the Asian II genotype and were closely related to isolates from Cuba 1981 and China 2015 epidemics, and the New Guinea C (NGC) 1944 prototype strain (Figure 1B). The topology of our phylogenetic tree based on partial NS5 sequences was similar to DENV-2 phylogeny based on the analysis of complete genomes^6^.

In the Americas, isolates from Cuba (1981), Venezuela (1994), and Mexico (1995) epidemics, detected in cases of dengue hemorrhagic fever, were closely related to Asian II genotype strains from Southeast Asia, including the NGC strain and strains from Thailand^7^. An NGC-like strain detected in Jamaica in 2007, causing mild dengue disease, was also grouped within the Asian II genotype^8^. Interestingly, the Asian II genotype caused meningitis in patients from Minas Gerais, in the southeast region of Brazil^9^, within the same timeframe as our sample collection. Our results confirmed Asian II genotype of DENV-2 circulation in Brazil, in addition to the prevalent Asian/American genotype.

Successive epidemics of dengue fever, with co-circulation of DENV-1, DENV-2, and DENV-4, have been reported in Sinop over the last 10 years. Sinop Forest Park is an area of permanent preservation, surrounded by urbanized neighborhoods and is visited by several city residents and tourists. The diversity of local fauna, which includes non-human primates, armadillos, foxes, and agoutis, serves as potential reservoirs for DENV^10^. Hence, sylvatic DENV cycle may have been established in the previous years, as occurred with Zika virus in another part of Brazil^11^. Our findings did not suggest a local spillback event of dengue virus; however, the presence of the virus in forest-collected mosquitoes raises important questions about the source of infection in mosquitoes and the presence of viruses in other forest animals.

The presence of the Asian II genotype of DENV-2 in the state of Mato Grosso in humans and mosquitoes is of paramount epidemiological importance. Our data not only confirm the circulation of this genotype in another state of Brazil other than Minas Gerais but also indicate the possibility of its spread to other regions of the country. Unlike the results reported in other studies, we also detected the Asian II genotype of DENV-2 in mosquitoes collected in the forest, which may be related to a spillback event of human dengue virus. Thus, continuous molecular surveillance of dengue virus in humans, vectors, and mammals in other regions of Brazil is required to further investigate the epidemiological status of this genotype.
